# ﻿*Ophiclypeus*, a new genus of Cardiochilinae Ashmead (Hymenoptera, Braconidae) from the Oriental region with descriptions of three new species

**DOI:** 10.3897/zookeys.1180.100106

**Published:** 2023-09-15

**Authors:** Ilgoo Kang, Mostafa Ghafouri Moghaddam, Michael J. Sharkey, Donald L. J. Quicke, Buntika A. Butcher, Christopher E. Carlton

**Affiliations:** 1 Department of Entomology, Louisiana State University Agricultural Center, 404 Life Sciences Building, Baton Rouge, LA, 70803 USA Louisiana State University Agricultural Center Baton Rouge United States of America; 2 Department of Entomology, College of Ecology and Environmental Science, Kyungpook National University, Sangju, Gyeongsangbuk-do, 37224, South Korea Kyungpook National University Sangju Republic of Korea; 3 Integrative Ecology Laboratory, Department of Biology, Faculty of Science, Chulalongkorn University, Phayathai Road, Pathumwan, Bangkok 10330, Thailand Chulalongkorn University Bangkok Thailand; 4 The Hymenoptera Institute, 41482 Alder Drive, Forest Falls, CA, 92339, USA The Hymenoptera Institute Forest Falls United States of America

**Keywords:** Ichneumonoidea, microgastroid complex, old world, parasitoid wasp, taxonomy

## Abstract

A new genus of the braconid subfamily Cardiochilinae, *Ophiclypeus***gen. nov.**, is described and illustrated based on three new species: *O.chiangmaiensis* Kang, **sp. nov.** type species (type locality: Chiang Mai, Thailand), *O.dvaravati* Ghafouri Moghaddam, Quicke & Butcher, **sp. nov.** (type locality: Saraburi, Thailand), and *O.junyani* Kang, **sp. nov.** (type locality: Dalin, Taiwan). We provide morphological diagnostic characters to separate the new genus from other cardiochiline genera. A modified key couplet (couplet 5) and a new key couplet (couplet 16) are provided with detailed images for Dangerfield’s key to the world cardiochiline genera to facilitate recognition of *Ophiclypeus***gen. nov.**

## ﻿Introduction

The Cardiochilinae Ashmead, 1900 is a small braconid subfamily with 237 described species in 18 genera (Kang 2022). Approximately half of all cardiochiline species were included in the two largest genera *Cardiochiles* Nees, 1819 and *Schoenlandella* Cameron, 1905, and most genera contain fewer than 10 species (Yu et al. 2016). In the Oriental region, 55 species in nine genera are recorded, which represents the highest species diversity and second highest generic diversity of Cardiochilinae in the world (Yu et al. 2016; Kang et al. 2020). Since 2000, seven genera and 22 new species have been described: one species of *Asiacardiochiles* Telenga, 1955 by Ahmad (2004); five species of *Austerocardiochiles* Dangerfield, Austin & Whitfield, 1999 by Ahmad (2004), Chen et al. (2004), and Long et al. (2019); one species of *Cardiochiles* by Ahmad (2004), three species of *Eurycardiochiles* Dangerfield, Austin & Whitfield, 1999 by Chen et al. (2004); nine species of *Hartemita* Cameron, 1910 by Ahmad (2004) and Long and van Achterberg (2011a, 2011b); two species of *Orientocardiochiles* Kang & Long, 2020 by Kang et al. (2020); and one species of *Schoenlandella* by Ahmad (2004).

Taiwan and Thailand are particularly poorly studied with regard to cardiochiline taxonomy. In Taiwan, six species in three genera of Cardiochilinae were recorded prior to the current study: *Cardiochilesalbopilosus* Szépligeti, 1902; *C.laevifossa* Enderlein, 1906; *C.philippensis* Ashmead, 1905; *Hartemitalatipes* Cameron, 1910; *H.townesi* Dangerfield & Austin, 1990, and *Schoenlandellaszepligetii* (Enderlein, 1906) (Dangerfield and Austin 1990; Chou 1995; Dangerfield et al. 1999; Yu et al. 2016). From Thailand, only one species, *C.philippensis* was recorded prior to the current study (Dangerfield and Austin 1995; Yu et al. 2016).

To contribute to the growing body of knowledge of Cardiochilinae in the Oriental region, we describe here a new genus, *Ophiclypeus* gen. nov., collected in Taiwan and Thailand, with three new species. We provide information based on morphology to diagnose the new taxa, as well as an identification key.

## ﻿Materials and methods

Specimens collected in Taiwan were borrowed from the Hungarian Natural History Museum (HNHM; Budapest, Hungary). The Thai specimen was borrowed from the Hymenoptera Institute (Forest Falls, CA, USA). Specimens were examined using stereomicroscopes (Leica® MZ75 and Fisher Scientific Model 420). Morphological terminology mainly follows Sharkey and Wharton (1997) and partly follows Dangerfield and Austin (1995). Terms for body sculpture follow Harris (1979). The following acronyms are used for morphological terms throughout: POL: distance between posterior ocelli, T1 (first metasomal tergum), T2 (second metasomal tergum), and T3 (third metasomal tergum).

Digital images were taken using a Visionary Digital BK Plus imaging system (Dun, Inc.) equipped with a Canon® EOS 5DS DSLR and were stacked using Zerene Stacker™ v. 1.04 (Zerene Systems LLC.). Adobe Photoshop® CS 6 and Photoshop® CC 2022 v. 23.0 (Adobe Systems, Inc.) were used to edit images and measure body characters. All measurements are given in millimeters, and numbers in parentheses in genus and species descriptions indicate the actual size of each body character. A distribution map of three species was generated using SimpleMappr (Shorthouse 2010) and was edited in the Adobe Photoshop® CC 2022 (Fig. 4).

## ﻿Results

### ﻿Taxonomic accounts

#### ﻿ Ophiclypeus

Taxon classificationAnimaliaHymenopteraBraconidae

Kang gen. nov.

E3CA6B87-C936-51F3-8A1F-3D4F6A2FEF28

https://zoobank.org/F1AB0281-3DEA-42F6-A9DF-55CC8F7F0401

##### Type species.

*Ophiclypeuschiangmaiensis* Kang, sp. nov.

##### Diagnosis.

**Figure 1.**
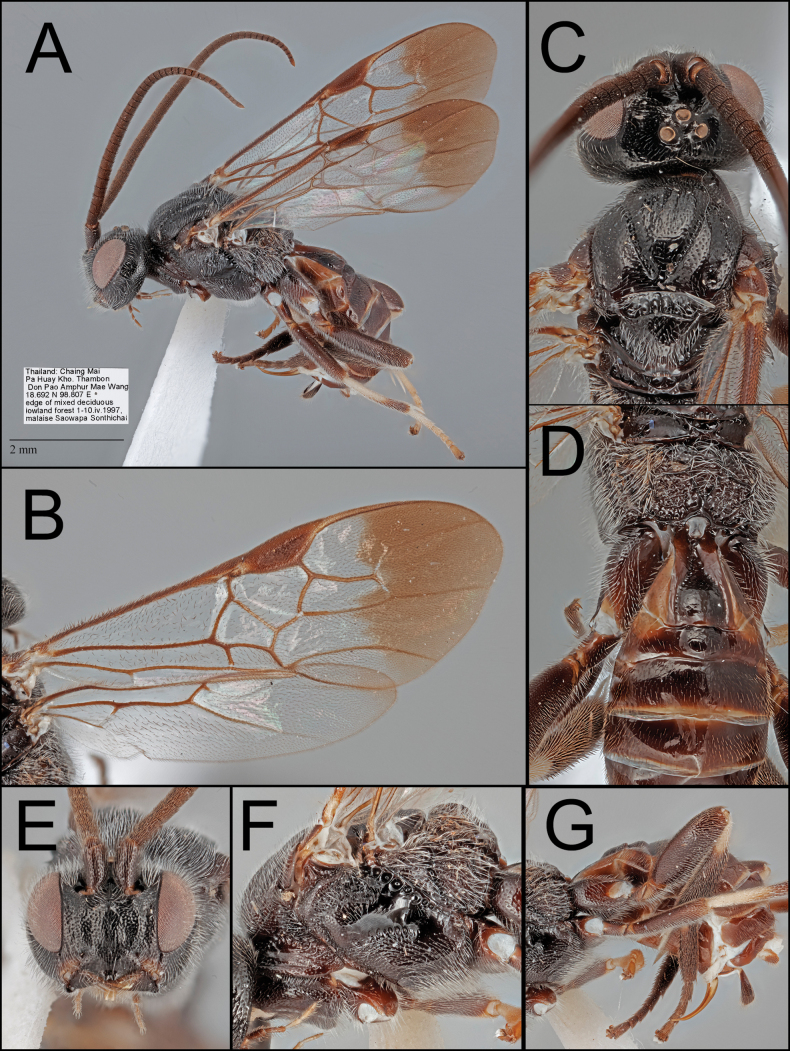
*Ophiclypeuschiangmaiensis* sp. nov. **A** lateral habitus **B** wings **C** dorsal head and mesoscutum **D** dorsal propodeum and metasoma **E** anterior head **F** lateral mesosoma **G** lateral metasoma.

**Figure 2.**
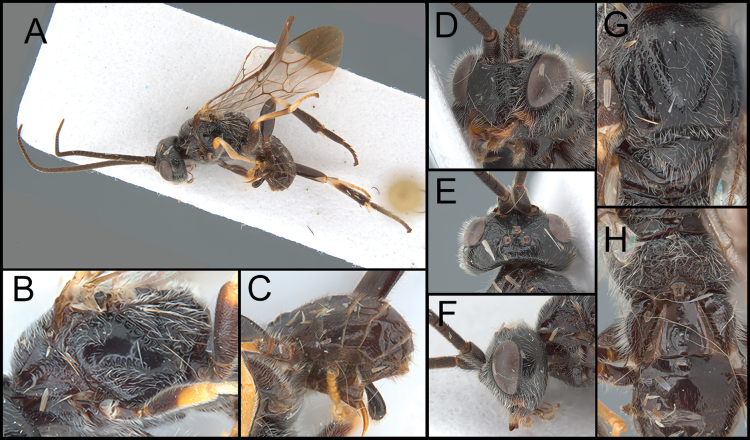
*Ophiclypeusdvaravati* sp. nov. **A** lateral habitus **B** lateral mesosoma **C** lateral metasoma **D** anterior head **E** dorsal head **F** lateral head **G** mesoscutum **H** dorsal propodeum and metasoma.

**Figure 3.**
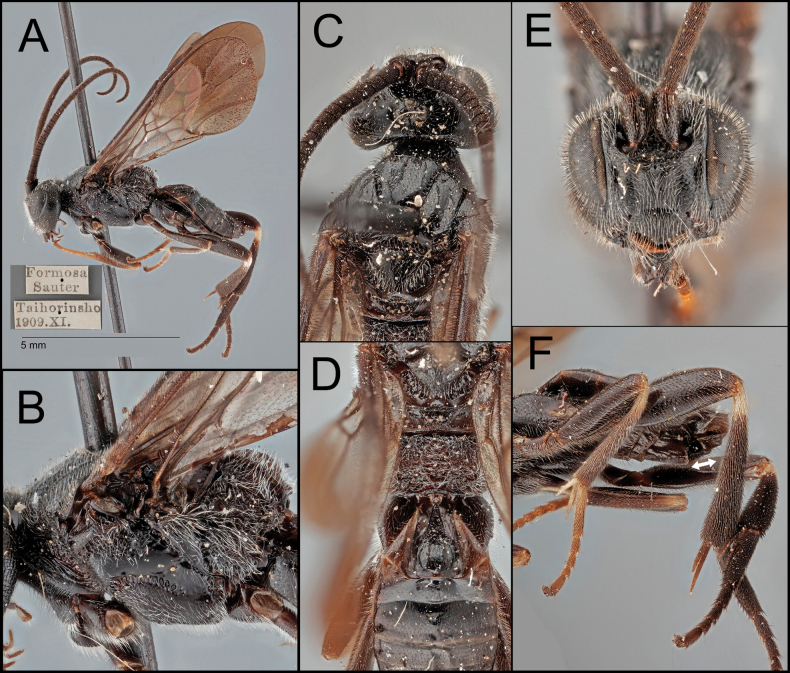
*Ophiclypeusjunyani* sp. nov. **A** lateral habitus **B** lateral mesosoma **C** dorsal head and mesoscutum **D** dorsal propodeum and metasoma **E** anterior head **F** lateral metasoma; arrows show ovipositor length.

Members of *Ophiclypeus* gen. nov. are similar to members of *Bohayella* Belokobylskij, 1987, *Hartemita* Cameron, 1910, *Hymenicis* Dangerfield, Austin & Whitfield, 1999, *Pseudcardiochilus* Hedwig, 1957, and *Retusigaster* Dangerfield, Austin & Whitfield, 1999 based on the following shared characters: ovipositor short, thick, and sharply downcurved; hypopygium short and not pointed apically. However, members of *Ophiclypeus* gen. nov. can be distinguished from the members of the other five genera by the following combination of characters: a narrow face (Figs 1E, 2D, 3E); long, dense interommatidial setae present (Figs 1E, 2D, 3E); clypeus with two sharp apical teeth (Figs 1E, 2D, 3E); hind tibia without expanded apex (Figs 2A, 3F); hind tarsus laterally expanded, but not enlarged as much as *Hartemita* (Figs 2A, 3F); claws pectinate; absence of 2–1A of hind wing (Figs 2A, 3F); propodeal areola completely developed (Fig. 3D); T1 1.1–1.3× longer than apical width (Figs 1D, 2H, 3D); short ovipositor sheath 0.3–0.6× longer than length of hind basitarsus; >0.2× longer than hind tibia (Figs 1G, 2C, 3F).

##### Description.

Body length 4.6–7.3 mm.

***Head*.** Antenna with 38–40 segments. Interantennal space with median carina. Eye densely setose, setae long. Gena extended ventro-posteriorly into moderate prominence. Clypeus with two sharp apical tubercles. Mandible bidentate. Maxillary palpus with 6 segments. Apical maxillary palpomere longer than fifth palpomere. Labial palpus with 4 segments. Galea short. Glossa short. Occipital carina absent.

***Mesosoma*.** Notauli entirely crenulate. Scutellar sulcus present, with five or six carinae. Postscutellar depression crenulate. Pronotum sculptured over most of its surface. Precoxal sulcus strongly crenulate and not reaching posterior margin. Epicnemial carina absent. Episternal scrobe straight. Metapleuron strongly sculptured.

***Legs*.** Basal spur on fore tibia 0.8–0.9× longer than length of basitarsus. Hind tibia without apical cup-like projection. Claws pectinate. Hind tarsus laterally expanded.

***Wings*.** Fore wing second submarginal cell trapezoid, longer than height; vein 1r absent; 3r present basally; 3RSb angled at basal third; stigma about 3.0× longer than wide medially. Hind wing 2r-m absent; 2–1A absent.

***Metasoma*.**T1 1.1–1.3× longer than its posterior width, posteriorly combined with laterotergite; area near Y-shaped suture anteriorly or entirely crenulate. T2 0.2–0.3× longer than its posterior width, with curved posterior margin. Hypopygium without median longitudinal fold. Protruded ovipositor sheath short and downcurved, 0.3–0.6× longer than length of hind basitarsus with long setae in apical half.

**Male.** Unknown.

##### Biology.

Unknown.

##### Distribution.

**Figure 4.**
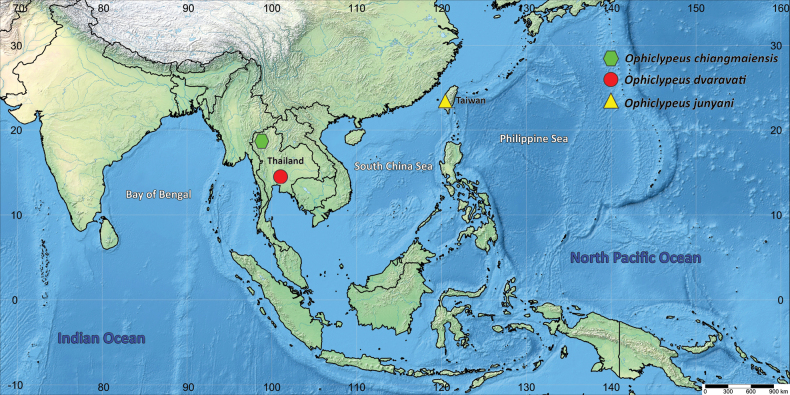
Distribution map of *Ophiclypeus* species.

Oriental region (Taiwan, Thailand) (Fig. 4).

##### Etymology.

The name for the genus refers to “shield with snake fangs”. From “ophi” (Greek for “snake”) and “clypeus” (Latin for “shield”). Gender: masculine.

##### Notes.

We modified the key to world genera of Cardiochilinae by Dangerfield et al. (1999) as follows:

**Table:** 

5(4)	**a.**T1 >3.0× longer than its posterior width	**6**
–	**b.**T1 1.1–2.0× longer than its posterior width	**16**
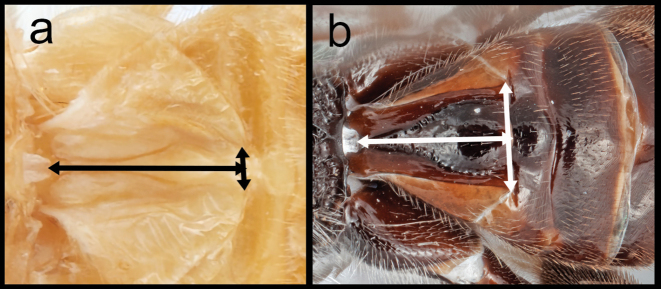		
16(5)	**a.** Eyes seemingly without interommatidial setae. **aa.** clypeus >2.5× longer than its height and without clypeal tubercles. **aaa.** 2–1A of hind wing present and extending halfway to wing margin	***Retusigaster* Dangerfield, Austin, & Whitfield, 1999**
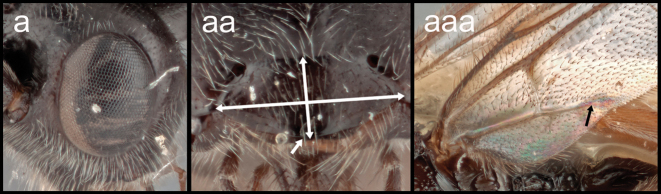		
–	**b.** Eyes with long and dense interommatidial setae; clypeus <2.0× longer than its height and with clypeal tubercles. **bb.** 2–1A of hind wing absent	***Ophiclypeus* gen. nov.**
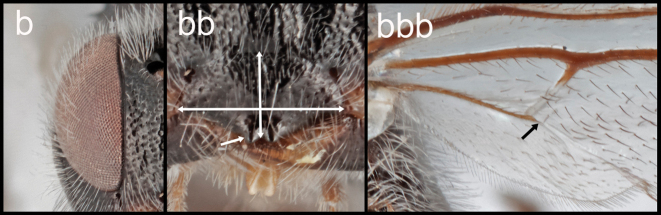		

### ﻿Illustrated key to *Ophiclypeus* species

**Table:** 

1	**A.** Fore femur entirely dark	***O.junyani* sp. nov.**
–	**B.** Fore femur apically pale	**2**		
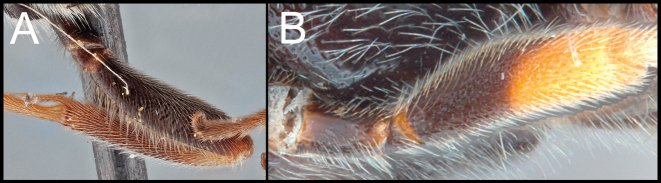
2(1)	**A.** Mesoscutum with stronger punctures. **AA.** Y-shaped suture entirely crenulate	***O.chiangmaiensis* sp. nov.**
–	**B.** Mesoscutum with weaker punctures. **BB.** Y-shaped suture entirely smooth	***O.dvaravati* sp. nov.**
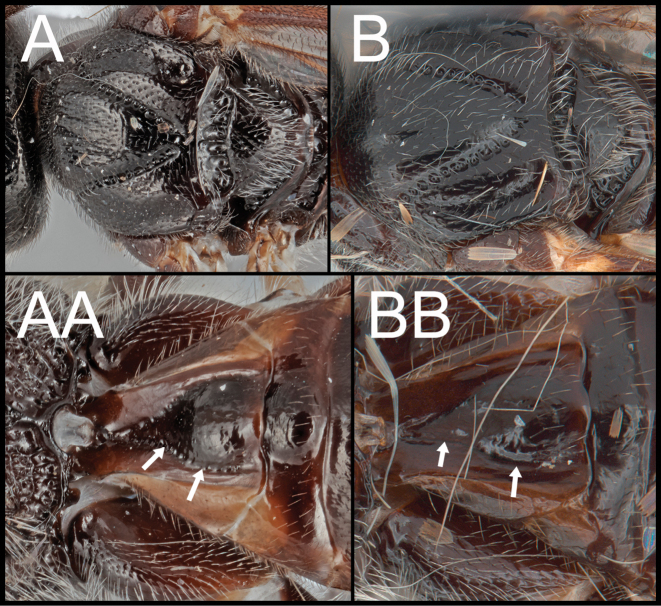		

#### ﻿ Ophiclypeus chiangmaiensis

Taxon classificationAnimaliaHymenopteraBraconidae

Kang sp. nov.

9EFF394E-1D8C-580E-8EFA-8C5DE6FA9247

https://zoobank.org/21A46417-2184-4469-8150-53DC9D32E5D5

Fig. 1A–G

##### Type material.

***Holotype*.** Thailand • ♀; Don Phao, Mae Wang, Pa Huay Kho, Chiang Mai, Thailand; 18.692°N, 98.807°E; iv.1997; Saowapa Sonthichai; collected in an edge of mixed deciduous lowland forest using Malaise trap. Will be deposited in Queen Sirikit Botanic Garden Entomology Collection (Chiang Mai, Thailand, QSBG).

##### Diagnosis.

Adult body size smaller than that of *O.junyani* sp. nov. Face with stronger punctures (Fig. 1E). Malar space 1.2× longer than basal width of mandible (Fig. 1E). Mesoscutum with stronger and larger punctures (Fig. 1C). Mesopleuron with stronger punctures (Fig. 1C). Fore femur apically pale. Apical fourth of fore wing infuscate (Fig. 1B). 3r of hind wing present basally (Fig. 1B). The ratio of propodeum (median length to width) = 0.7 (Fig. 1D). Propodeal areola narrow and spindle-shaped (Fig. 1D). Inner space of Y-shaped suture entirely smooth (Fig. 1D). Y-shaped suture entirely crenulate (Fig. 1D).

##### Description.

Body 5.5 mm.

***Head*.** Antenna with 38 segments. Face width 1.2× longer than its height (1.02:0.83). Width of anterior ocellus 0.8× longer than POL (0.15:0.18). Median width of eye about 0.8× longer than the median width of gena in lateral view (0.29:0.36). Clypeus 1.9× longer than its height (0.67:0.35). Malar space 1.2× longer than basal width of mandible (0.24:0.20).

***Mesosoma*.** Scutellar sulcus with five carinae. Pronotum ventrally carinate, posteriorly crenulate. Mesopleuron dorsally rugulose, medially smooth, ventrally punctate (evenly punctured entirely). Metapleuron crenulate medially and rugulose anteriorly and posteriorly. Propodeum 0.7× longer than its median width (0.67:0.96), strongly rugulose; median areola 2.1× longer than its maximum width (0.53:0.25) and spindle-shaped.

***Legs*.** Basal spur on fore tibia 0.9× longer than length of basitarsus (not measured using images). Basal spur on mid tibia 0.9× longer than length of basitarsus (0.59:0.64). Basal spur on the hind tibia 0.7× longer than length of basitarsus (0.62:0.88).

***Wings*.** Fore wing 5.5 mm; second submarginal cell trapezoid, 2.8× longer than height (1.05:0.38); pterostigma about 2.8× longer than wide medially (1.08:0.38).

***Metasoma*.**T1 1.2× longer than its posterior width (0.79:0.64), separated with lateral tergum by suture anteriorly and by color posteriorly; Y-shaped suture entirely crenulate; inner space of Y-shaped suture entirely smooth. T2 0.3× longer than its posterior width (0.33:1.34), with curved posterior margin, 0.7× longer than T3 (0.33:0.50). T3 0.3× longer than its posterior width (0.50:1.48). Protruded ovipositor sheath 0.5× longer than length of hind basitarsus (0.47:0.88), with long setae at apical half.

***Color*.** Body mostly black or dark brown except for the following, which are pale ivory or white: area between lateral clypeus and dorsal mandible; apical and penultimate maxillary palpomeres; glossa; apical fore femur; entire fore tibia, fore tarsus, and mid tarsus; basal mid tibia and hind tibia; tibial spurs; T1 laterally; ovipositor. Wings hyaline basally and infuscate at apical fourth. Pterostigma mostly dark except for base and apex. Body color is similar to a pattern of *O.dvaravati* sp. nov. but possessing brighter metasoma and several whitish leg parts.

**Male.** Unknown.

##### Biology.

Unknown.

##### Distribution.

*Ophiclypeuschiangmaiensis* sp. nov. is known from Don Pao, Mae Wang, Chiang Mai, Thailand (Fig. 4).

##### Etymology.

This species is named after the collecting site, “Chiang Mai Province”.

##### Notes.

The first author attempted to obtain molecular data from a specimen of *O.chiangmaiensis* sp. nov. collected in 1997 but failed, and there was no attempt to acquire molecular data from a specimen of *O.dvaravati* sp. nov. collected in 2016. In the future research, molecular analyses based on newly collected specimens and portions of existing museum specimens will be helpful in placing *Ophiclypeus* gen. nov. into a broader phylogenetic context with other cardiochilines.

#### ﻿ Ophiclypeus dvaravati

Taxon classificationAnimaliaHymenopteraBraconidae

Ghafouri Moghaddam, Quicke & Butcher sp. nov.

C9408DD6-D40E-540C-A499-7DEF0A261593

https://zoobank.org/1705521A-4233-4AE2-A781-C32F4D481247

Fig. 2A–H

##### Type material.

***Holotype*.** Thailand • ♀; Chulalongkorn University campus, Cham Phak Phaeo, Kaeng Khoi District, Saraburi, Thailand; 14°31’44.72”N, 101°1’57.25”E; 25.vi.2016; P. Kerkig; collected in an edge of secondary forest near to a large reservoir using Malaise Trap. The type is deposited in the Collection of the Insect Museum, Chulalongkorn University Museum of Natural History (Bangkok, Thailand, CUMZ).

##### Diagnosis.

Face with weaker punctures than *O.chiangmaiensis* sp. nov. (Fig. 2D). Malar space 0.9× longer than basal width of mandible (Fig. 2D). Mesoscutum with weak punctures (Fig. 2G). Mesopleuron dorsally smooth, ventrally weakly punctate (Fig. 2B). Fore femur apically pale orange. Apical quarter of fore wing infuscate (Fig. 2A). 3r of hind wing absent (Fig. 2A). Propodeal areola nearly a rhombus and its ratio (median length to width) = 0.4 (Fig. 2H). Y-shaped suture of T1 entirely smooth; inner space of Y-shaped suture of T1 slightly sculptured entirely (Fig. 2H).

##### Description.

Body length 4.6 mm.

***Head*.** Antenna with 38 segments. Face width slightly less than its height. Malar space 0.9× longer than basal width of mandible. Width of anterior ocellus 0.7× longer than POL. Median width of eye about 1.0× longer than the median width of gena in lateral view. Clypeus 1.5× longer than its height.

***Mesosoma*.** Scutellar sulcus bearing five or six carinae. Pronotum medially crenulate, postero-dorsally carinate. Mesopleuron dorsally smooth with sparse setae, medially smooth without setae, ventrally finely punctate. Propodeum 0.4× longer than its median width, strongly rugulose; median areola 1.5× longer than its maximum width.

***Legs*.** Basal spur on the fore tibia 0.8× longer than length of basitarsus. Basal spur on the mid tibia 0.9× longer than length of basitarsus. Hind tibia without apical cup-like projection; basal spur on the hind tibia 0.7× longer than length of basitarsus; hind claw with four teeth.

***Wings*.** Fore wing 4.2 mm; second submarginal cell 3.0× longer than height; pterostigma about 2.8× longer than wide medially. 3r of hind wing basally absent.

***Metasoma*.**T1 1.1× longer than its posterior width, separated with lateral tergum by weakly different color; Y-shaped suture entirely smooth; inner space of Y-shaped suture entirely slightly sculptured. T2 0.2× longer than its posterior width, with curved posterior margin, 0.6× longer than T3. T3 0.4× longer than its posterior width. Protruded ovipositor sheath 0.6× longer than length of hind basitarsus.

***Color*.** Body mostly black or dark brown except for the following, which are pale orange or yellow: area between lateral clypeus and dorsal mandible; apical and penultimate maxillary palpomeres; glossa; apical fore femur; entire fore tibia, fore tarsus, and mid tarsus; basal mid tibia and hind tibia; tibial spurs; T1 laterally; ovipositor. Wings hyaline basally and infuscate at apical fourth. Stigma mostly dark except for base and apex. The color pattern is similar to a pattern of *O.chiangmaiensis* sp. nov., but much darker metasoma and without whitish leg parts.

**Male.** Unknown.

##### Biology.

Unknown.

##### Distribution.

*Ophiclypeusdvaravati* sp. nov. is known from Saraburi, Thailand (Fig. 4).

##### Etymology.

This species is named after Dvaravati, an ancient Mon kingdom from the 7^th^ to 11^th^ century, which was located in what is now central Thailand.

#### ﻿ Ophiclypeus junyani

Taxon classificationAnimaliaHymenopteraBraconidae

Kang sp. nov.

5D522A60-9301-5825-B7C2-F617DFAD02AE

https://zoobank.org/C967E257-A3AB-4D32-88A1-4EFBF786AEDC

Fig. 3A–F

##### Type materials.

***Holotype*.** Formosa (Taiwan) • ♀; Taihorinsho (Dalin, Chiayi); xi.1909; H. Sauter. ***Paratype*** Same as holotype except for Takao (Kaohsiung); 1907. Both specimens are deposited in HNHM.

##### Diagnosis.

Adult body size larger than that of *O.chiangmaiensis* sp. nov. Face with weaker punctures than *O.chiangmaiensis* sp. nov. (Fig. 3E). Malar space 1.8× longer than basal width of mandible (Fig. 3E). Mesoscutum with weak punctures (Fig. 3C). Mesopleuron dorsally with weaker punctures than *O.chiangmaiensis* sp. nov. (Fig. 3B). Fore femur entirely darker (Fig. 3A). Apical half of fore wing infuscate (Fig. 3A). The ratio of propodeum (median length to width) = 0.6 (Fig. 3D). Propodeal areola broad and nearly a rhombus (Fig. 3D). Inner space of Y-shaped suture sculptured anteriorly (Fig. 3D). Y-shaped suture anteriorly crenulate and posteriorly smooth (Fig. 3D).

##### Description.

Body length 7.3 mm.

***Head*.** Antenna with 40 segments. Face width 1.1× longer than its height (1.05:0.93). Width of anterior ocellus 0.8× longer than POL (0.15:0.18). Median width of eye about 0.7× longer than the median width of gena in lateral view (0.44:0.58). Clypeus 1.9× longer than its height (0.70:0.36). Malar space 1.8× longer than basal width of mandible (0.29:0.16).

***Mesosoma*.** Scutellar sulcus bearing five or six carinae. Pronotum medially carinate, posteriorly crenulate. Mesopleuron dorsally rugulose, medially smooth, ventrally punctate (anteriorly with stronger punctures). Propodeum 0.6× longer than its median width (0.79:1.44), strongly rugulose; median areola 1.8× longer than its maximum width (0.66:0.37) and nearly rhombus-shaped.

***Legs*.** Basal spur on the fore tibia 0.8× longer than length of basitarsus (0.48:0.60). Basal spur on the mid tibia 0.9× longer than length of basitarsus (0.68:0.80). Hind tibia without apical cup-like projection; basal spur on the hind tibia 0.6× longer than length of basitarsus (0.74:1.18); hind claw with four teeth.

***Wings*.** Fore wing 6.5 mm; second submarginal cell 2.8× longer than height (1.21:0.43); stigma about 3.1× longer than wide medially (1.33:0.43).

***Metasoma*.**T1 1.3× longer than its posterior width (1.00:0.79), separated with lateral tergum by color; Y-shaped suture anteriorly crenulate and posteriorly smooth; inner space of Y-shaped suture anteriorly sculptured. T2 0.2× longer than its posterior width (0.36:1.58), with curved posterior margin, 0.6× longer than T3 (0.36:0.61). T3 0.4× longer than its posterior width (0.61:1.65). Protruded ovipositor sheath 0.3× longer than length of hind basitarsus (0.38:1.18).

***Color*.** Body mostly black or dark brown except for the following, which are pale yellow or white: apical mandible, basal tibiae, fore and mid tarsi, fore and mid tibial spurs. Wings mostly clear at basal half and mostly infuscate at apical half. Pterostigma entirely dark.

**Male.** Unknown.

##### Biology.

Unknown.

##### Distribution.

*Ophiclypeusjunyani* sp. nov. is known from Dalin and Kaohsiung, Taiwan (Fig. 4).

##### Etymology.

Named in honor of Mr Junyan Chen, PhD candidate in the Department of Entomology at LSU AgCenter, for his help with the first author’s research. Mr Chen has fond memories of a trip to Dalin, Taiwan.

## Supplementary Material

XML Treatment for Ophiclypeus

XML Treatment for Ophiclypeus chiangmaiensis

XML Treatment for Ophiclypeus dvaravati

XML Treatment for Ophiclypeus junyani

## ﻿Additional information

### Author ORCIDs

Ilgoo Kang https://orcid.org/0000-0002-8501-1758

Mostafa Ghafouri Moghaddam https://orcid.org/0000-0002-1942-9689

Michael J. Sharkey https://orcid.org/0000-0001-6201-7340

Donald L. J. Quicke https://orcid.org/0000-0003-4471-6775

Buntika A. Butcher https://orcid.org/0000-0002-0541-0709

### Data availability

All of the data that support the findings of this study are available in the main text or Supplementary Information.
